# Impact of Shipping Transit Time on Central Laboratory Processing of Total Colony Forming Units (CFU) and Staphylococcus aureus Detection

**DOI:** 10.7759/cureus.80590

**Published:** 2025-03-14

**Authors:** Randy W Loftus, Franklin Dexter, Jeremiah R Brown

**Affiliations:** 1 Anesthesiology and Perioperative Medicine, Mayo Clinic Rochester, Rochester, USA; 2 Anesthesia, University of Iowa, Iowa City, USA; 3 Epidemiology, Dartmouth Geisel School of Medicine, Lebanon, USA

**Keywords:** central clinical laboratory, colony forming units (cfu), detection, s aureus, shipment stability, transit time

## Abstract

Background: Central laboratory processing of anesthesia work area reservoir samples is used to improve infection control measures. Reservoir samples returning ≥ 100 colony forming units (CFU) and *Staphylococcus aureus* (*S. aureus) *detection are monitored to identify improvement targets. The impact of sample shipment time under ambient conditions on these meaningful outcomes has not been characterized. Such insight could help to further optimize feedback that has been proven to generate substantial reductions in surgical site infections. In this study, we aimed to assess the impact of ambient shipping conditions on patient intravenous stopcock sample CFU ≥ 100 and *S. aureus* detection because stopcock contamination is repeatedly associated with increased patient mortality.

Methods: We conducted a retrospective analysis involving seven geographically dispersed hospitals over a 4.2-year (October 1, 2018 to December 31, 2022) study period. We chose geographically dispersed sites considering variation in ambient shipping conditions and time. Stopcocks sampled at the end of surgery were shipped to a central laboratory, plated to sheep’s blood agar, incubated for 24hr at 36°C, CFU/mL quantified, and distinct isolates assessed by colony morphology, Gram stain, simple rapid tests (e.g., coagulase, oxidase, lactose fermentation, catalase), and selective growth medium.

Results: A total of 969 stopcock samples were analyzed. The percentage of stopcocks with CFU ≥ 100 was stable following sample collection from days 3 to 32 (odds ratio (OR) 1.0086/day, 95% confidence interval (CI) 0.9868-1.0309/day) and from sample kit preparation from days 3 to 143 (OR 1.0044/day, 95% CI 0.9991-1.0099/day). *S. aureus* detection decreased beyond 14 days from the period of collection during the surgical procedure (P = 0.0024; OR 0.83, 95% CI 0.21-0.71).

Conclusions: When utilizing a central laboratory for processing anesthesia workspace reservoir stopcock set samples, there is stability of ≥ 100 CFU for up to 32 days from collection and up to 143 days from kit preparation. *S. aureus* detection remains stable for up to 14 days. Therefore, when monitoring stopcock contamination to provide feedback, samples should be processed within 14 days from their collection. Anticipated shipment times should be considered by sample collection personnel to ensure optimal sample yield.

## Introduction

Monitoring of total colony forming units (CFU) and *Staphylococcus aureus* (*S. aureus)* transmission among anesthesia workspace reservoirs proven to be associated with bacterial transmission and surgical site infection (SSI) development can be used to optimize basic preventive measures and in turn, to generate substantial reductions in *S. aureus *transmission and SSIs [1,2]. Monitored reservoirs map to basic preventive measures [3,4]. The adjustable pressure-limiting valve and agent dial of the anesthesia machine map to between-case, post induction, and terminal cleaning of the anesthesia environment. Anesthesia provider (attending and in-room provider) hands map to provider hand hygiene before and after patient care. Patient skin sites (nares, axilla, and groin) map to preoperative decolonization and intraoperative asepsis. The patient intravenous stopcock (stopcock) at case end maps to injection port and syringe tip disinfection [3-5]. Reservoirs that return ≥ 100 CFU [6,7] and/or for which there is *S. aureus* detection [8-10] indicate improvement targets for the respective preventive measure [1,2]. Feedback to hospitals regarding improvement targets is used to optimize basic preventive measures to improve patient safety [1,2].

A National Institutes of Health (NIH) study (BASIC Trial, R01AI155752) was designed to determine the best methodology for national dissemination of this approach of proven efficacy [1] and effectiveness [2] in attenuating *S. aureus* transmission and SSIs [1,2]. For the study, anesthesia work area reservoir isolates obtained from geographically dispersed hospitals in the United States were shipped to a central laboratory for processing to report total CFU and pathogen detection. Participating hospital sites were instructed to ship collected samples within one week to allow for a total time of 14 days from collection to plating to nutrient media. Because observed shipment delays have exceeded 30 days, and the swab transport system used for sample collection (Amies transport medium, Copan Diagnostics, Murrieta, CA) ensures pathogen survival for up to three weeks at room temperature [11], variability in ambient shipping conditions and transit times may have impacted culture yield. Further insight into the potential impact of shipment delays and associated variability in exposure to ambient shipping conditions is necessary to inform the BASIC trial results. This information will also be useful for future epidemiological field studies where logistical considerations such as shipment delays are an important consideration [1,2,11]. 

Prior work has begun to assess these considerations. In one study, Amies transport medium was inoculated separately with 10 distinct methicillin-resistant *S. aureus* (MRSA) and vancomycin-resistant *Enterococcus* (VRE) clinical isolates, and the specimens were stored at room temperature or 4°C. Pathogen stability was confirmed for both storage conditions for up to 14 days [11]. Another study assessed the stability of eight clinical *S. aureus *isolates under ambient laboratory conditions (room temperature) for up to three weeks. While the authors confirmed pathogen detection up to 21 days, culture yield dropped substantially after day 18 [12]. Shipment time has been shown to provide substantial contributions to overall sample processing time which may also impact culture yield [13]. This prior work is limited by simulated laboratory conditions evaluating only a small subset of clinical pathogens [11,12] and theoretical considerations [13].

In the current study, we utilized samples collected from geographically dispersed hospitals in the United States to assess the real-world impact of shipment transit time and variation in ambient shipping conditions on culture yield. We focused our analysis on patient intravenous stopcock (stopcock) samples returning ≥ 100 CFU and/or positive for *S. aureus, *clinically relevant outcomes associated with major bacterial pathogen detection and SSIs, respectively [6,7,9]. In addition, stopcock contamination is repeatedly associated with increased all-cause patient mortality [5,14], and stopcocks are appropriately without exposure to surface disinfection chemicals that may impact pathogen viability in subsequent culture because they are in direct continuity with the intravascular space. This would not be true for other measured reservoirs such as environmental surfaces. We analyzed total CFU to allow consideration for pathogens extending beyond *S. aureus* and VRE [11], and we extended analysis of transit times beyond 21 days [12]. 

Given that temperature variation is an important consideration for assessment of samples shipped to a central laboratory from geographically dispersed hospitals, we leveraged prior work evaluating pathogen stability despite temperature variation in the laboratory setting [11] to establish our primary hypothesis that stopcock CFU ≥ 100 and *S. aureus* detection would remain stable for up to 14 days from their initial collection. We explored the potential association of stopcock *S. aureus* isolate detection with other major Gram-negative [15,16] and/or enterococcal pathogens [17].

## Materials and methods

Study design

This was a retrospective analysis of patient stopcock samples obtained from seven geographically dispersed hospitals in the United States over 4.2 years (October 1, 2018 to December 31, 2022) with a total follow-up period of 606 days. The University of Iowa Institutional Review Board determined that this study involving analysis of deidentified data of microbiological samples did not meet the regulatory definition of human subjects’ research and, hence, IRB review is not required. All seven hospitals collected data to monitor bacterial transmission to improve basic preventive measures in the anesthesia work area [1,2]. All stopcock samples that were sent to the central laboratory (RDB Bioinformatics, Coralville, IA) for processing were considered for the analysis. Dr. Dexter conducted the formal analysis. The three authors of this study collaborated virtually and were all involved in the design, data acquisition, interpretation, manuscript preparation, and approval of the final manuscript.

Inclusion and exclusion criteria

Inclusion Criteria

Operating room environments involving the surgical care of adult or pediatric patients undergoing surgery requiring the administration of general anesthesia according to usual practice and placement of a peripheral intravenous catheter (stopcock). Most observations included a case-pair, the stopcocks for two patients undergoing care sequentially in the same operating room. Collected stopcock samples had to be shipped and exposed to ambient shipping conditions, positive for ≥ 1 CFU after processing, and with complete and accurate information regarding the date of surgery.

Exclusion Criteria

Stopcock samples unexposed to ambient shipping conditions, resulting in zero CFU after processing, incomplete information regarding surgery date, and inaccurate date of surgery recorded (e.g., listed after the date of laboratory plating).

Data collection

Sterile swab collection systems (ESwab, Copan Diagnostic Inc., Corona, CA) were sent to each hospital via collection kits for stopcock culture at the end of the surgical procedure. The injection port primarily used by the anesthesia provider(s) was sampled. For open lumen devices, the entire internal lumen was sampled. For closed, disinfectable stopcocks, the surfaces contacting syringe tips were sampled. In both scenarios, swabs were rotated 360° 10 times to culture [1]. Collected samples were then shipped to a central laboratory (RDB Bioinformatics, Coralville, Iowa) via FedEx ground under ambient shipping conditions. Hospital sites were instructed to ship samples within one week of collection. Shipped samples remained under ambient laboratory conditions until processing with the date of processing recorded, including the initial plating to 5% sheep’s blood agar (SBA). After vortexing for 15 seconds, the 1 mL of Amies transport medium was transferred to an 85 mm SBA plate and incubated for 24hr at 35°C. Total CFU/mL were then quantified by counting four quadrants, and plates with ≥ 500 CFU were considered too numerous to count. Distinct isolates were assessed by colony morphology, Gram stain, simple rapid tests (e.g., coagulase, oxidase, lactose fermentation, catalase), selective growth medium, and analytical profile indexing [3,5,8,15-17]. Each stopcock sample was linked to surgical case characteristics (e.g., surgical date) and microbiological data (CFU and *S. aureus* detection) via a unique barcode [5].

Outcomes

Studying the impact of transit time, the primary outcome of interest was stopcock samples returning ≥ 100 CFU [6,7], and the secondary outcome of interest was detection of ≥ 1 *S. aureus* isolate(s), both among stopcock sets contaminated with ≥ 1 CFU [5,14]. We explored detection of other major bacterial pathogens (e.g., Gram-negative and enterococcal) when *S. aureus* was detected.

Statistical analysis

We did not perform a statistical power analysis because we planned a priori to use every available stopcock sample for the study period, meeting inclusion criteria. The association between the study outcomes and transit time was analyzed using linear logistic regression (Stata v18.5, StataCorp, College Station, TX). If P ≥ 0.05, we considered the 95% confidence intervals to be narrow if the contrasts of the predictive margins were ≤ 10% over 13 days from surgery to SBA, and 42 days from kit preparation to SBA, the medians of the actual return durations.

## Results

A total of 2,660 stopcock samples were evaluated with 969 included in the final analysis. Excluded samples included lack of exposure to ambient shipping conditions (N=24), zero CFU after processing (N=1,501), incomplete recording of the surgical date (N=159), and inaccurate documentation of the surgical date (N=7). Characteristics of the cases and microbiological samples of the included stopcock samples are listed in Tables 1, 2.

**Table 1:** Characteristics of the 969 studied stopcocks; each sampled at the end of the patient’s surgical case. From the incidence of *Staphylococcus aureus* contamination (8.5%) and the incidence of Gram-negative contamination (15%), the expected incidence of both would be 1.2%, where 1.2% = 8.5% × 15%. By Fisher’s exact test, the observed incidence of both (2.9%) is significantly greater than expected by chance, P < 0.0001.

Characteristic	Summary measure of the 969 stopcocks
At least 100 colony forming units	618 (64%)
First case of the day in the operating room	540 (56%)
Second case of the day in the operating room	429 (44%)
Distinct operating rooms	85
Distinct hospitals’ cases	7
Cases from hospital with most cases	370 (38%)
Cases from hospital with second most cases	310 (32%)
Winter, December 21 through March 19	232 (24%)
Spring, March 20 through June 20	140 (14%)
Summer, June 21 through September 21	349 (36%)
Fall, September 22 through December 20	248 (26%)
Days from sample collection on the day of surgery to plating to 5% sheep's blood agar (SBA) was ≥ 3 days	945 (98%)
Days from sample collection on the day of surgery to plating to 5% SBA was 3 to 32 days	865 (89%)
*S. aureus* pathogen contamination	82 (8.5%)
Methicillin-resistant *S. aureus *pathogen contamination	12 (1.2%)
Gram-negative(including *Pseudomonas *spp.) pathogen contamination	143 (15%)
*S. aureus* and Gram-negative pathogen contamination	28 (2.9%)
Coagulase-negative *Staphylococcus *pathogen contamination	332 (34%)

**Table 2:** Days from sample collection and collection kit preparation to plating on 5% sheep's blood agar (SBA). Days from sample collection on the day of surgery to plating to SBA refers to delays generated by the hospital sites in shipping the samples combined with the duration of shipping. Samples were maintained under ambient shipping and laboratory conditions until they were plated to SBA and incubated overnight. Shipping of collection kit for hospital use to plating refers to the duration spanning from when the kits containing the collection materials were prepared and sent to the hospital and their return to the central laboratory for processing.

	Days	Percentile of the 969 stopcock samples
Sample collection on day of surgery to plating	2	2.6
	3	5
	4	10
	8	25
	13	50
	18	75
	30	90
	32	91.5
	48	93
	211	95
Shipping of collection kit for hospital use to plating	15	5
	23	10
	33	25
	42	50
	66	75
	143	89.2
	150	90
	317	95

**Figure 1:**
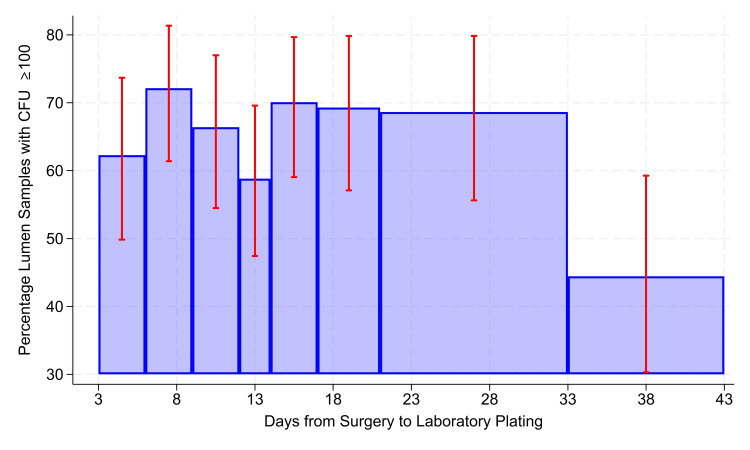
Association between colony forming units (CFU) ≥ 100 and days from surgery to plating to 5% sheep's blood agar (SBA). From a total of 969 samples (Table 1), 945 were included in this analysis because 24 samples were not exposed to ambient shipping conditions; rather, they were transported directly to the laboratory via use of a currier. The error bars show 99% two-sided Clopper-Pearson confidence intervals, 99% used because of the multiple bars. All bars in the chart include ≥ 102 stopcocks in the denominator and ≥ 32 stopcocks in the numerator, with one exception. The bar shown on the horizontal axis as extending from 33 to 43 days, shown as such only for clarity of presentation, summarizes the leftover counts of 80 stopcocks with observed times from collection on the day of surgery to initial plating to SBA from 33 to 490 days (median time of 224 days). With an alternative threshold for each bar involving a denominator ≥ 75 CFU, the numerators did not meet the criteria of ≥25 CFU (i.e., far wider confidence intervals).

There were up to 490 days from sample collection to SBA. The percentage of stopcocks with CFU ≥ 100 was stable following collection from days 3 to 32 (Figure 1).

**Figure 2:**
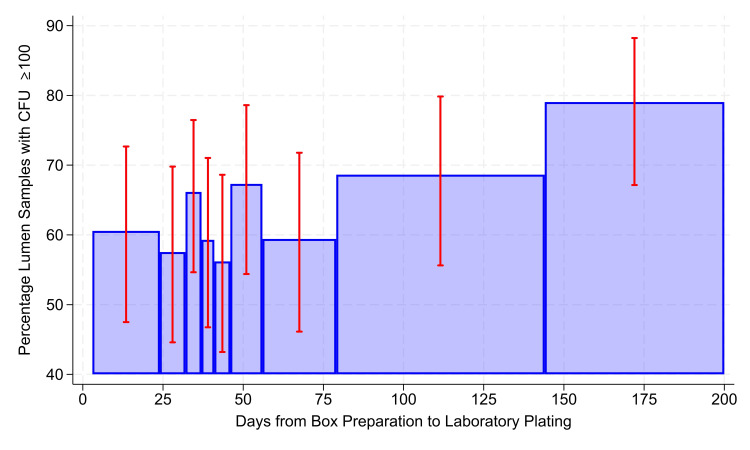
Association between colony forming units (CFU) ≥ 100 and days from kit preparation to plating to 5% sheep's blood agar (SBA). This figure depicts the association between stopcock CFU ≥ 100 and the number of days from preparation of the collection kit to plating to 5% sheep's blood agar (SBA). The error bars show 99% two-sided Clopper-Pearson confidence intervals, with 99% used because of the multiple bars. All bars in the chart include ≥ 101 stopcocks with CFU ≥ 1 in the denominator and 32 stopcocks in the numerator, with one exception. The bar summarizing 144 to 200 days (horizontal axis) includes leftover counts with observations up to 585 days. When the alternative threshold for each bar was a denominator ≥ 75 CFU, the numerators did not meet the criteria of ≥ 25 CFU (i.e., far wider confidence intervals).

The odds ratio was approximately 1.0086 per day, not differing significantly from 1.00 (P = 0.44). The 95% confidence interval was narrow, 0.9868 to 1.0309 per day. While the interval from three (2.6th percentile) to 32 days (91.5th percentile) had a quartile deviation of four days (Table 2), the observations ≥ the 92nd percentile had a quartile deviation of 76 days, making estimates too imprecise for use (Figure 1). The percentage of stopcocks with CFU ≥ 100 was stable when kit preparation to SBA was 3-143 days (Table 3, Figure 2).

**Table 3:** Logistic regression analyses for the dependent variable of ≥ 100 CFU among contaminated stopcock sets. CFU: Colony forming units

Independent variable	Independent variable (50th, 25th, 75th percentiles)	Dependent variable overall percentage (sample size)	Estimated odds ratio per day	95% confidence interval
Days surgery to laboratory testing	3 to 32 days (12, 85, 16)	67% (578/865)	1.0086	0.9868-1.0309
Days box and media preparation to laboratory testing	3 to 141 days (40, 32, 54)	62% (535/864)	1.0045	0.9991-1.0099

The odds ratio was approximately 1.0044 per day, not differing significantly from 1.00 (P = 0.10). The 95% confidence interval was also narrow, from 0.9991 to 1.0099 per day (Table 3).

**Figure 3:**
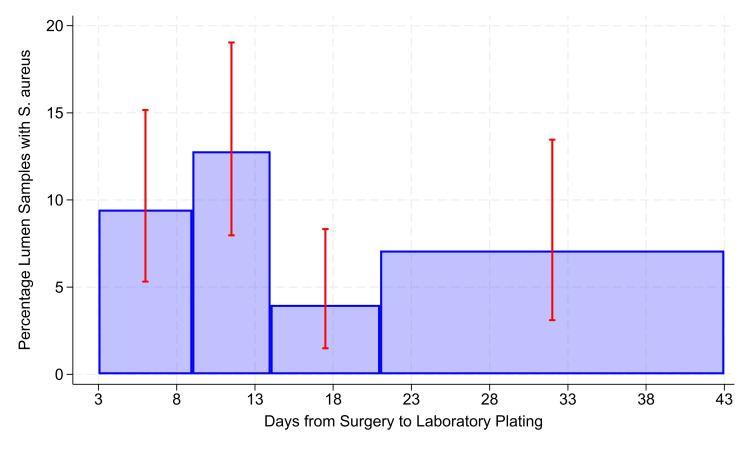
Days from surgery to laboratory plating. The error bars show 99% two-sided Clopper-Pearson confidence intervals, with 99% being used because of the multiple bars. All bars in the chart include ≥ 250 stopcocks with ≥ 100 CFU (denominator) and ≥ 10 stopcocks with *Staphylococcus aureus* detected, with one exception. For clarity of presentation, the bar presented as extending from 21 to 43 days, shown on the horizontal axis and matching Figure 1, included 182 stopcocks with times from collection on the day of surgery to plating spanning 21 to 490 days (median of 31 days). Reducing the sample sizes from ≥ 250 to ≥ 225 or to ≥ 200 stopcocks did not change categories and included the threshold of 14 days, matching the earlier study [11]. When the alternative threshold for each bar was a denominator ≥ 150 stopcocks, the numerators included values of five (i.e., far wider confidence intervals).

There were N = 865 stopcocks with CFU ≥ 1 when the interval from surgery to plating ranged from 3 to 32 days (Table 3). The overall rate of stopcock contamination with *S. aureus* was 8.1% (71/865). There was a significant decrease in *S. aureus* detection when the interval of surgery to plating exceeded 14 days (P = 0.0024; odds ratio 0.83, 95% confidence interval 0.21 to 0.71; Figure 3).

The risk ratio equaled 0.41, where 0.41 = (13/306)/(58/559). Confirmed pathogen contamination rates among stopcocks with ≥ 1 CFU were as follows: *S. aureus* 8.5% (82/969), MRSA 1.2% (12/969), Gram-negative pathogens including *Pseudomonas *spp. 15% (143/969), *S. aureus* and Gram-negative pathogens in the same stopcock sample 2.9% (28/969), and coagulase-negative *Staphylococcus* 34% (332/969) (Table 1). From the incidence of *S. aureus *contamination (8.5%) and the incidence of Gram-negative contamination (15%), the expected incidence of both would be 1.2%, where 1.2% = 8.5% × 15%. By Fisher’s exact test, the observed incidence of their coincidence (2.9%) was significantly greater than expected by chance, P < 0.0001.

Among the 969 stopcocks studied with ≥ 1 CFU (Table 1), CFU ≥ 100 had an 8.2% incidence of *S. aureus* (51/618), and CFU < 100 had an 8.8% incidence (31/351). The estimated risk ratio equaled 0.93, P = 0.76. When all 2,660 stopcocks were used, stopcocks with CFU ≥ 100 had an 8.5% incidence of *S. aureus* contamination (60/704) whereas stopcocks with CFU < 100 had a 1.8% incidence (34/1932) of *S. aureus* contamination. The estimated risk ratio equaled 4.84, where 4.84 = 8.5%/1.8%, P < 0.0001 (see Discussion section).

## Discussion

A centralized laboratory has been used to monitor anesthesia workspace reservoirs to identify improvement targets in basic preventive measures [1-4]. Important improvement targets include reservoirs returning ≥ 100 CFU and/or resulting in *S. aureus* detection [6-11]. Feedback regarding these improvement targets can generate substantial reductions in *S. aureus* transmission and SSIs [1,2]. National implementation (BASIC Trial (R01AI155752) of this improvement strategy is underway given its proven efficacy [1] and effectiveness [2]. The trial methodology employs a central laboratory for reservoir sample processing where substantial shipment delays have been observed. In this study we have leveraged these shipment delays to gain insight into the real-world impact of transit time and associated ambient conditions on clinically relevant culture yields [1-11], samples returning ≥ 100 CFU and *S. aureus* detection. We found that CFU ≥ 100 remained stable for up to 32 days from collection at the time of the surgical procedure, supporting our primary hypothesis that there is sample stability for at least 14 days following collection on the day of surgery [11]. In a secondary analysis we found that *S. aureus* detection was also stable for up to 14 days [11]. Thus, bacterial cultures collected in the field should be processed within 14 days of their collection to optimize yield.

Substantial delays in shipment of samples from geographically dispersed hospital sites may impact microbiological results due to changes in transit time that are associated with variability in exposure to ambient environmental conditions (e.g., temperature and humidity) and total processing time [11-13]. Prior work simulated these conditions in the laboratory to assess the impact on *S. aureus* stability [11,12]. In one study, patient-derived, clinical *S. aureus* and VRE strains were evaluated for up to 14 days under different temperature conditions (room temperature and 4°C), and CFU were quantified and compared to baseline cultures. Defining stability as ≤ 3-log reduction [11], the authors concluded that there was stability for culture of these pathogens for up to 14 days. In another study, stability of clinical* S. aureus* isolates was confirmed for up to 18 days when subjected to ambient laboratory conditions (room temperature) [12]. Taken together, laboratory data suggest that *S. aureus *isolates can remain stable in Amies transport medium for two weeks [11,12]. However, neither study [11,12] assessed the impact of real-world variability in ambient shipping conditions or transit times derived from shipping to a central laboratory from multiple geographic regions. In addition, the impact of transit times extending beyond 21 days [12] has not been assessed, an important limitation given that transit time has been shown to contribute substantially to total sample processing time, which may also impact culture yield [13].

The current study adds to this body of literature by examining the impact of transit time on total CFU from patient stopcock samples from multiple, geographically dispersed hospitals in the United States that were subjected to substantial variation in ambient conditions. We excluded negative stopcocks from the analysis because they have no growth on which to assess the impact of shipping conditions, and their inclusion would inappropriately bias the results towards lack of viability. As a secondary aim we evaluated the impact on *S. aureus* isolate detection, where survival of ≥ 1 CFU is required (categorically distinct from evaluations defining stability as a ≤ 3-log reduction [11]), and we explored the parallel detection of *S. aureus* with other major bacterial pathogens. These were clinically relevant assessments because total CFU correlate with increased risk of major bacterial pathogens [6,7], and *S. aureus* detection can lead to transmission among anesthesia workspace reservoirs which is associated with increased risk of SSI development [9]. In our study of seven unique hospitals and 85 distinct operating rooms, we found that over 60% of contaminated stopcocks returned ≥ 100 CFU and that this magnitude of contamination was evenly distributed by season. We found that CFU ≥ 100 remained stable from day 3 to 32 following collection. This was true from sample kit preparation and from date of surgery to SBA, findings that support both reagent and bacterial culture stability, respectively. These results support reagent stability for many months (day 3-143, approximately five months). This latter finding is helpful because it can help to guide decisions on when to return unused sample collection kits. These results support stability in the measurement of transmission of intravascular devices.

Consistent with prior literature, we also found that stopcock *S. aureus* detection was stable for 14 days [11,12]. Whereas prior work assessed single strains and defined stability by a ≤ 3-log reduction from the starting inoculum [11], we assessed detection, which requires only 1 CFU. In our analysis of detection, we observed that *S. aureus* detection may increase by day 21. There is a decline from day 14 to 20, but there may be a return to baseline by day 21 (Figure 3). This could relate to several factors. It may be that anaerobic conditions that ensue by day 14 result in quorum sensing and biofilm formation [18]. This could help to explain in part why most SSIs occur within the first two weeks (median onset postoperative day 9-10) [19] and that delayed, chronic infections are often persistent and more difficult to treat; the surviving organisms were more fit [20]. Interestingly, the gap from day 14 to 21 may indicate a period of vulnerability that could in future study be exploited to enhance SSI prevention, a period where adaptive mechanisms such as quorum sensing [18] or unknown mechanisms have not yet occurred. One could reasonably hypothesize that use of physiological steroids to enhance innate cellular immunity during this window, whereas reported by Yeager et al., “release of cortisol could prime immune cells for an augmented response to a subsequent immune challenge,” may help to prevent SSIs [21]. Future study could also compare single nucleotide variants [22] among organisms that are detected greater than 21 days from collection vs. those that are detected much earlier to identify variants that associate with survival. Those variants could then be validated in subsequent isolate populations [23] and potentially exploited for new drug therapy. Alternatively, because oxygen can diffuse through the primary receptacle made of plastic [24], and microbes use oxygen during metabolism [25], that by day 21 the impact of a shift of oxygen from outside to inside the primary receptacle occurs, enhancing bacterial growth. This premise could readily be tested in a future study by simply measuring dissolved concentrations of oxygen stratified by transit time. Thus, while our limited sample size did not provide statistical significance, the observed trend as shown in Figure 3 warrants further study that is likely to be made possible with completion of the BASIC trial.

We found in our exploratory analysis that when *S. aureus* is detected there is increased detection of other major bacterial pathogens such as Gram-negative pathogens (e.g. *Pseudomonas *spp.), where ≥ two or more such pathogens occurred with a probability that exceeded significantly that which would occur by chance alone. This finding can help to support the current approach to monitoring of anesthesia workspace reservoirs where *S. aureus* is the sentinel pathogen [1,2], why a response to such monitoring can help to reduce all-cause SSIs [1,2], why *S. aureus* transmission is associated with increased risk of all-cause SSI development [9], and why stopcock contamination is repeatedly associated with increased patient mortality and linked to infection via molecular techniques [5,14]. Given that we show substantial magnitude of stopcock contamination in this study and an alarming incidence, it is apparent that proven methods for improved vascular care should be implemented aggressively [26,27]. These include improved design and handling of both peripheral and central devices, with a number needed to treat of only 11 to prevent one contamination event and 16 to prevent one case of HAI and/or phlebitis. More extensive use of a centralized laboratory to provide feedback via the approach described in this study may help to achieve this goal [1,2].

Limitations

Our study may have been limited by the lack of inclusion of stopcock samples returning zero CFU. As stopcocks could have zero CFU either because of no contamination or bacterial death, stopcocks with zero CFU were excluded from our primary analysis. As we aimed to assess the impact of transit time on survival, the inclusion of stopcocks with zero CFU would be confounding. Therefore, the 1,501 stopcocks with zero CFU were appropriately excluded for Figures 1, 3. As explained in the results, among the 969 stopcocks studied with CFU ≥ 1 (Table 1), the CFU ≥ 100 had no greater incidence of *S. aureus*, with a risk ratio of approximately 0.93, P = 0.76. Thus, the CFU ≥ 100 shown in Figure 1 were not temporally associated with the *S. aureus* contamination shown in Figure 3. However, again from the results, when stopcocks with zero CFU were included, then stopcocks with CFU ≥ 100 more often had *S. aureus* contamination, with risk ratio approximately 4.84, P < 0.0001. These heterogeneous observations are jointly consistent with prior methodology [6]. Embedded in this logical premise is a comparison to no transmission, where stopcock CFU are zero, not a comparison among cases of transmission. As such, we appropriately focused on the outcomes of CFU ≥ 100 and on *S. aureus* detection for CFU ≥ 1 when considering the impact of transit time. An additional limitation of our work is the retrospective design. Studies involving intentional contamination of stopcock sets would not be allowed given the association with increased patient mortality [5,14], so our work provides the most reasonable next step toward assessing the real-world impact of ambient shipping conditions on clinically relevant outcomes measured in a central laboratory. As a sensitivity analysis, a future study could involve contamination of stopcock sets that are not used for patient care with a known inoculum. Contaminated samples could then be shipped from the same geographic regions after simulated shipment delays to the central laboratory for processing using the described methodology and the impact on culture yield assessed. However, this approach would not account for real-world assessment of stability of measured contamination in the clinical environment or real-world logistical constraints. The results of our retrospective, real-world analysis are strengthened by their agreement with laboratory findings [11,12]. We were unable to confirm stability of stopcock CFU ≥ 100 across the studied range. However, it may be that an even longer transit time is stable. Even though some observations of transit time extended to 490 days, there was too much variability to draw meaningful conclusions beyond the 92nd percentile. A larger sample size will be needed. Our sample size for *S. aureus* isolates among contaminated stopcock sets was limited. As this was our secondary aim, this secondary analysis should be interpreted with caution, and a future study should be conducted to further validate the study results. 

## Conclusions

The stability of the clinically relevant outcomes of anesthesia work area stopcock CFU ≥ 100 or *S. aureus* detection when exposed to ambient shipping conditions has not been previously assessed. Following assessment of these outcomes involving multiple, geographically dispersed hospitals in the United States, we report that stopcock CFU ≥ 100 remained stable for 32 days following their initial collection during surgery, up to 143 days from kit preparation, and that *S. aureus* detection declined after 14 days. While future study should determine the cause of shipment delays in order that they can be prevented to optimize culture yield, processing samples within 14 days is suitable
